# Pharmaceutical immunoglobulin G impairs anti-carcinoma activity of oxaliplatin in colon cancer cells

**DOI:** 10.1038/s41416-021-01272-6

**Published:** 2021-02-09

**Authors:** Yuru Shang, Xianbin Zhang, Lili Lu, Ke Jiang, Mathias Krohn, Stephanie Matschos, Christina Susanne Mullins, Brigitte Vollmar, Dietmar Zechner, Peng Gong, Michael Linnebacher

**Affiliations:** 1 Department of General Surgery, Molecular Oncology and Immunotherapy, Rostock University Medical Center, Schillingallee 69, 18057 Rostock, Germany; 2 Institute for Experimental Surgery, Rostock University Medical Center, Schillingallee 69a, 18059 Rostock, Germany; 3 Shenzhen University General Hospital & Shenzhen University Clinical Medical Academy, Xueyuan Road 1098, 518055 Shenzhen, China; 4 Present Address: Shenzhen University General Hospital & Shenzhen University Clinical Medical Academy, Xueyuan Road 1098, 518055 Shenzhen, China

**Keywords:** Preclinical research, Molecular medicine, Colon cancer

## Abstract

**Background:**

Recent evidence proves that intravenous human immunoglobulin G (IgG) can impair cancer cell viability. However, no study evaluated whether IgG application benefits cancer patients receiving chemotherapeutics.

**Methods:**

Influence of pharmaceutical-grade human IgG on the viability of a series of patient-derived colon cancer cell lines with and without chemotherapeutic intervention was determined. Cell death was analysed flow cytometrically. In addition, the influence of oxaliplatin and IgG on the ERK1/2-signalling pathway was evaluated by western blots.

**Results:**

We evaluated the effects of pharmaceutical IgG, such as PRIVIGEN^®^ IgG and Tonglu^®^ IgG, in combination with chemotherapeutics. We did not observe any significant effects of IgG on tumour cell viability directly; however, human IgG significantly impaired the anti-tumoral effects of oxaliplatin. Primary cancer cell lines express IgG receptors and accumulate human IgG intracellularly. Moreover, while oxaliplatin induced the activation of ERK1/2, the pharmaceutical IgG inhibited ERK1/2 activity.

**Conclusions:**

The present study demonstrates that pharmaceutical IgG, such as PRIVIGEN^®^ IgG and Tonglu^®^ IgG, can impair the anti-carcinoma activity of oxaliplatin. These data strongly suggest that therapeutic IgG as co-medication might have harmful side effects in cancer patients. The clinical significance of these preclinical observations absolutely advises further preclinical, as well as epidemiological and clinical research.

## Background

Oxaliplatin is the third generation of platinum anticancer agents. Compared to other platinum derivatives, such as cisplatin and carboplatin, the amine groups of oxaliplatin are replaced by diaminocyclohexane resulting in faster and more effective DNA synthesis inhibition.^1–4^ Preclinical studies demonstrated synergistic anticancerous effects of oxaliplatin in combination with 5-fluorouracil in colon cancer cells.^3,5^ Clinically, addition of oxaliplatin to 5-fluorouracil and leucovorin (FOLFOX regimen) can significantly improve the survival of colon cancer patients^6,7^ and oxaliplatin-containing regimen became a therapy standard for management of colon cancer.^8,9^

Several proteins and mechanisms are involved in the anticancerous activity of oxaliplatin. For example, the oxaliplatin-induced DNA damage leads to the expression of excision repair cross-complementation group 1 (ERCC1), which triggers cancer cell resistance to oxaliplatin.^10^ In addition, oxaliplatin can induce the activation of extracellular signal-regulated kinases 1 and 2 (ERK1/2), a master protein of the mitogen-activated protein kinase (MAPKs) pathway.^11^ Blocking ERK1/2 activity significantly promotes oxaliplatin cell toxicity.^11^ However, some studies reported that the anti-carcinoma activity of platinum agents is dependent of the ERK1/2 activity.^12–15^ These data suggest an ambivalent function of ERK1/2, and the relationship between ERK1/2 activity and oxaliplatin still has to be fully evaluated.

Immunoglobulin G (IgG) is the most common type of antibody produced and secreted by plasma B cells. In clinical practice, IgG supplementation especially benefits patients with inflammatory diseases.^16^ Since inflammation has been accepted as an emerging hallmark of cancers, more and more clinical trials try to evaluate the benefit of anti-inflammatory strategies in cancer management.^17^ In addition, it is a fact that cytotoxic chemotherapy impairs the immune system up to the point of severe immune deficiency.^18^ To compensate the latter, IgG supplementation might be indicated for some cancer patients. At present, there is scarce preclinical or clinical evidence proving that IgG can impair cancer growth.^19–22^ Notably, there is no study evaluating the influence of IgG supplementation on the outcome of chemotherapeutic interventions.

Currently, most colon cancer cells used for tissue culture are more than 40 years old. For example, the SW480 [SW480] (ATCC^®^ CCL-228™), a widely used colon cancer cell line, was isolated from a 50-year-old male colon cancer patient in 1976.^23^ A variety of colon cancer cell lines are cross-contaminated by the SW480 cell line.^24^ In addition, most classical colon cancer cell lines have been passaged hundreds of times. The overpassaged cells might present distinct characteristics from their original cancer tissues and generate erroneous results.^25^ Thus, in order to deliver more clinically relevant results, we investigated if and how pharmaceutical-grade IgG affects the anti-carcinoma activity of oxaliplatin in colon cancer cells using a series of novel patient-derived colon cancer cell lines.^26^ In addition, we evaluated if the ERK1/2 signal transduction pathway was involved in the interactions between IgG and oxaliplatin.

## Methods

### Patient-derived colon cancer cells and cell culture

The clinical and pathological characteristics of the colon cancer patients included in this study are summarised in Supplementary Fig. 1, and the process of establishing patient-derived cell lines has been reported in previous studies, for example Mullins et al.^26^ All patients signed the written informed consent and the procedures were approved by the Ethics Committee of the University Hospital of Rostock (reference number II HV 43/2004 and A 45/2007) in accordance with the declaration of Helsinki. Primary cell lines, directly established from human fresh tumour tissues, are indicated with the prefix HROC (Hansestadt Rostock colorectal cancer) and the ID number of the patient, such as HROC18. Cell lines, which were derived from patient-derived xenograft (PDX), are denoted with additional indices. For example, T indicating the times of transfer in an immunodeficient mouse, and M the ID number of mice, such as HROC131 T0 M3; Met indicates metastatic tumour. HROC18, HROC131 T0 M3 (HROC131), HROC147Met (HROC147), HROC277Met2 (HROC277Met), HROC277 T0 M1 (HROC277), HROC278Met T2 M2 (HROC278), HROC285 T0 M2 (HROC285), HROC370, HROC374, HROC46 T0 M1 (HROC46), HROC50 T1 M5 (HROC50) and HROC87 T0 M2 (HROC87) were cultured in DMEM/F12 (1:1) (PAN-Biotech, Aidenbach, Germany, code P04-41500) supplemented with 10% foetal bovine serum (FBS). Non-T cells (containing appr. 60% B cells) as well as T cells were obtained from a healthy donor after separation using the Pan T Cell Isolation Kit (Miltenyi, Bergisch-Gladbach, code 130-096-535) and were short-term cultured in IMDM. All cells grew in a humidified 5% CO_2_ incubator at 37 °C.

### Chemical drugs and pharmaceutical immunoglobulin

The oxaliplatin was obtained from Sun Pharmaceutical Industries Limited (Mumbai, India) and the PRIVIGEN^®^ Immune Globulin Intravenous (human), 10% Liquid, was purchased from CSL Behring (King of Prussia, Pennsylvania). The Tonglu^®^ Human Immunoglobulin was obtained from Tonglu (Hefei, China), and the IgG1, a main subclass of PRIVIGEN^®^ IgG, was purchased from R&D Systems (Minneapolis, USA, code 110-HG-100). In addition, PD98059 (code 9900) and U0126 (code 9903S), two inhibitors of mitogen-activated protein kinases 1 and 2 (MEK1/2), were obtained from Cell Signaling Technology (Danvers, USA).

### Evaluating cell viability

To evaluate the cell viability, 1.2 × 10^4^ HROC cells per well were seeded into a 96-well flat- bottom plate. After 24 h, cells were treated by appropriate vehicle (sham), oxaliplatin, PRIVIGEN^®^ IgG or the combination therapy with the concentrations defined in the figure legends. After 5 days, crystal violet assay was performed with 0.2% staining solution (AppliChem, Darmstadt Germany, code 131762) and the optical density was measured at 570 nm with the help of a Tecan Infinite 200 Microplate Reader (Tecan, Männedorf, Switzerland).

### Evaluating cell death

In order to evaluate cell death, 2 × 10^5^ cells per well were plated into a 6-well plate. After treating the cells with sodium chloride (sham), 6.25 µM oxaliplatin, 5 mg/mL PRIVIGEN^®^ IgG, 5 mg/mL Tonglu^®^ IgG, 100 ng/mL IgG1 or the combination therapy for 48 h, the cells were stained by FITC-conjugated Annexin V (ImmunoTools, Friesoythe, Germany, code 31490013) and propidium iodide (PI, AppliChem, code A2261). The percentage of dead cells was determined with the help of the FACSCalibur device (Becton Dickinson, New Jersey, USA) and CellQuest™ Pro software (Becton Dickinson) using the following formula: 100% × (Annexin V^+^ PI^–^ + Annexin V^+^ PI^+^ + Annexin V^−^ PI^+^) cells/total cells. In order to investigate if inhibition of ERK1/2 activity could impair the oxaliplatin-induced cell death, HROC277 and HROC285 cells were pretreated with dimethyl sulfoxide (sham), 50 µM PD98059 or 10 µM U0126 for 1 h.

### Evaluation of tumour cell-derived IgG and the expression of Fcγ receptors

In order to assess the levels of tumour cell-derived IgG in colon cancer cells, 4 × 10^5^ HROC cells were fixed with 400 μl of 2% formafix for 15 min. Subsequently, these cells were washed with 400 μl of PBS and were incubated with 400 μl of 1× buffer P (100× buffer P: 0.5 ml FBS, 5 ml 1% saponin, 5 ml 0.1 M HEPES and 39.5 ml PBS). After 10 min, the cell suspension was centrifuged at 300 × *g* for 8 min and cells were resuspended in 200 μl 1× buffer P and incubated with 5 μl FITC-conjugated goat anti-human IgG heavy- and light-chain antibody (Bethyl Laboratories, Montgomery, USA, code A80-119F) and 4 μl PE-conjugated mouse anti-human CD19 (ImmunoTools, code 21270194). After 30 min, the fluorescence intensity was measured and analysed as described before. With this staining method, intracellular as well as membrane-bound IgG is determined. T-cell-depleted human lymphocytes containing 60% B cells served as positive, human T cells as negative controls. In addition, to determine if the recombination of IgG genes occurred in the HROC cells, the DNA was extracted and the BIOMED-2 protocol was applied.^27^ In order to evaluate the expression of the FcγRI (CD64), FcγRII (CD32) and FcγRIII (CD16), three receptors of IgG,^28^ 5 × 10^5^ HROC cells were collected and the following antibodies (all from ImmunoTools) were used for extracellular staining: mouse anti-human CD64 (code 21270641), mouse anti-human CD32 (code 21330321) and mouse anti-human CD16 (code 21279161).

### p44/42 (ERK1/2) MAP kinase siRNA transfection

To inhibit the phosphorylation of ERK1/2, 5 × 10^5^ HROC285 cells per well were seeded in a 6- well plate for 24 h. Then the cells were transfected with 100 nM ERK1/2 siRNA (Cell Signaling Technology, code 6560) or appropriate control siRNA (Cell Signaling Technology, code 6568) with the help of Lipofectamine 3000 (Thermo Fisher Scientific, Waltham, USA, code L3000001). Forty-eight hours later, these cells were treated for 36 h by 6.25 µM oxaliplatin and cell death or ERK1/2 activity was determined.

### Western blot

In order to measure the levels of ERCC1 and ERK1/2, 5 × 10^5^ HROC277, HROC285, HROC370 or HROC374 cells per well were seeded in a 6-well plate, and treated by sham, 6.25 µM oxaliplatin, 5 mg/mL PRIVIGEN^®^ IgG, 5 mg/mL Tonglu^®^ IgG, 100 ng/mL IgG1 or the combination therapy indicated in the figure legends. After 36 h or 48 h, cells were harvested and western blot was performed as previously described using mouse anti-ERCC1 (Santa Cruz Biotechnology, Texas, USA, code sc-17809, dilution: 100×), mouse anti-ERK1/2 (R&D Systems, code MAB15761, dilution: 500×), rabbit anti-phospho-ERK1/2 (p-ERK1/2, R&D Systems, code MAB1018, dilution: 1000×) and mouse anti-β-actin antibody (Sigma-Aldrich, code A5441, dilution: 20,000×), followed by the secondary antibodies: peroxidase-linked anti-rabbit (Cell Signaling Technology, code 7074, dilution: 5000–10,000×) or anti-mouse antibodies (Sigma-Aldrich, code A9044, dilution: 20,000–60,000×). Proteins were visualised as previously described.^29^

### Data presentation and statistical analysis

Data were presented as mean ± standard deviation (SD) or box plot, and the statistical test of each figure was described in the figure legend. The normality and variance were determined by Shapiro–Wilk normality test and by Levene’s median equal variance test, respectively. To determine the significance of differences, the data were analysed by one-way analysis of variance with Holm–Sidak’s post hoc test, Kruskal–Wallis one-way analysis of variance on Ranks with Tukey’s post hoc test, Student *t* test or Mann–Whitney *U* test. In order to reduce the chances of type I errors, the Bonferroni correction was performed. All statistics were performed by Sigmaplot 12.0 (Systat Software, San Jose, CA, USA).

## Results

### Characteristics of patient-derived colon cancer cell lines used

Twelve low-passage cell lines established from eleven colon cancer patients were used in this study (Supplementary Fig. 1). Patients HROC147, HROC285, HROC370 and HROC374 were still alive, while patients HROC131, HROC277, HROC278 and HROC46 died at the time point of follow-up (May/2020). The progression-free survival of these patients varied from 0 months to 60 months, and the overall survival varied from 16 months to 95 months (Supplementary Fig. 1).

### Pharmaceutical IgG impairs anti-carcinoma activity of oxaliplatin

In order to evaluate if pharmaceutical IgG might have an effect on HROC cells, we first investigated if pharmaceutical-grade IgG influences the viability of colon cancer cells. Commercially available and clinically used pooled human normal IgG (i.e. PRIVIGEN^®^) was added to HROC cell lines (Supplementary Fig. 2). PRIVIGEN^®^ IgG did not significantly influence the viability of any of the cancer cell lines, independent of whether cells were cultured under nutrition-rich (10% FBS) or starved (0% FBS) conditions (Supplementary Fig. 2).

**Fig. 1:**
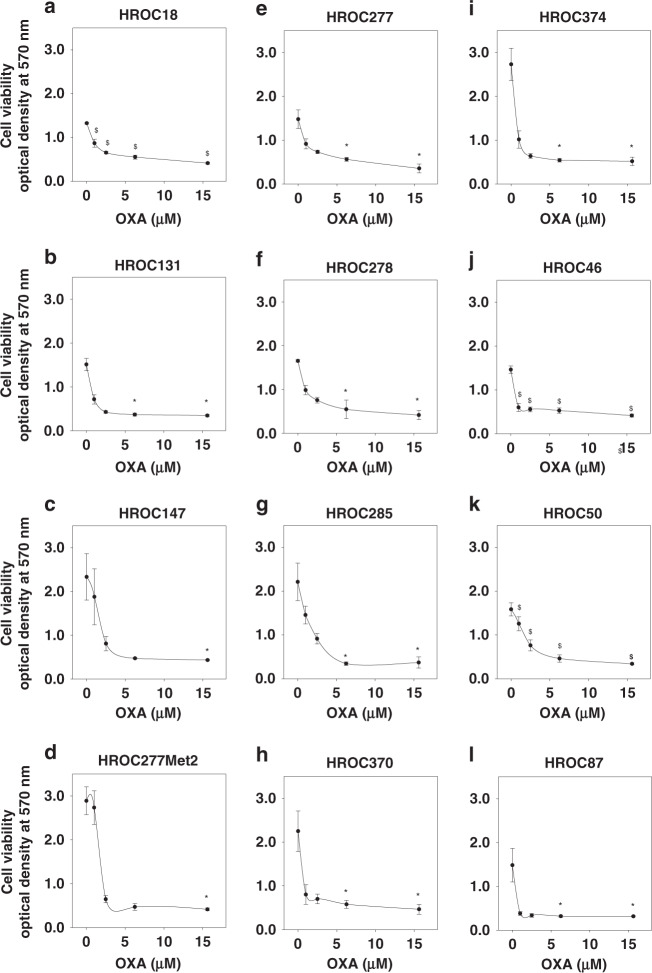
Oxaliplatin inhibits cell viability. HROC18 (**a**), HROC131 T0 M3 (**b**), HROC147Met (**c**), HROC277Met2 (**d**), HROC277 T0 M1 (**e**), HROC278Met T2 M2 (**f**), HROC285 T0 M2 (**g**), HROC370 (**h**), HROC374 (**i**), HROC46 T0 M1 (**j**), HROC50 T1 M5 (**k**) and HROC87 T0 M2 (**l**) were treated with appropriate vehicle, 1 µM, 2.5 µM, 6.25 µM or 15.6 µM oxaliplatin (OXA) for 5 days. Oxaliplatin significantly inhibited the cell viability. $ indicates *P* < 0.001, which was determined by one-way analysis of variance with Holm–Sidak’s post hoc test; * indicates *P* < 0.05, which was determined by Kruskal–Wallis one-way analysis of variance on Ranks with Tukey’s post hoc test. For (**e**), (**g**), (**h**) and (**i**), *N* = 6; for the other cell lines, *N* = 4.

**Fig. 2:**
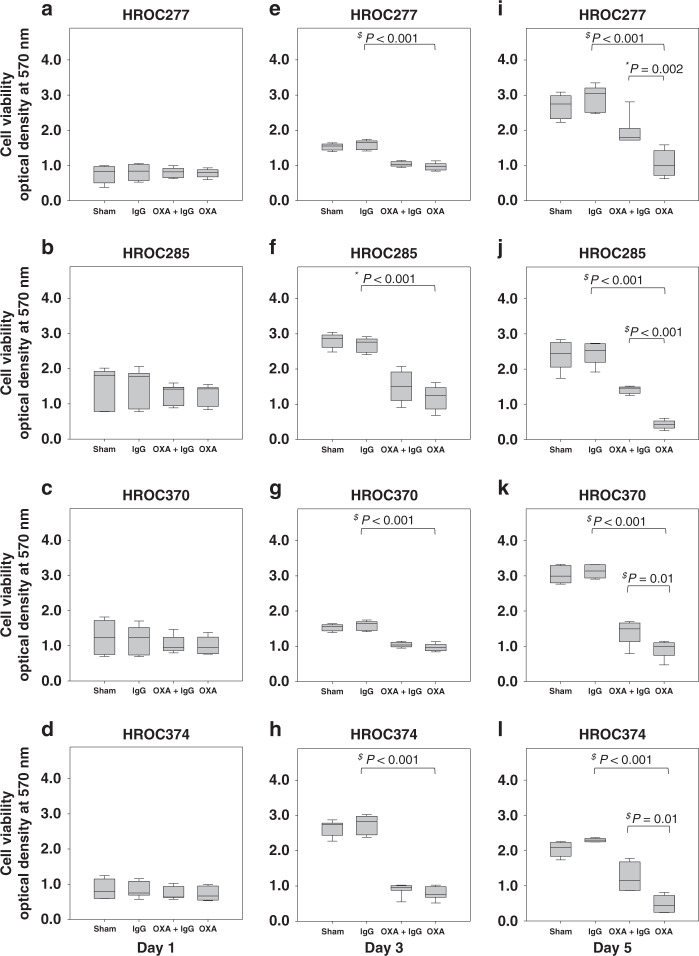
PRIVIGEN^®^ IgG impairs the cell-toxic effects of oxaliplatin. The indicated colon cancer cell lines were treated with sham, 6.25 µM oxaliplatin, 1.25 mg/mL, 2.50 mg/mL and 5 mg/mL PRIVIGEN^®^ IgG, or the combination therapy (oxaliplatin plus IgG) for 1 day (**a**), 3 days (**b**) or 5 days (**c**). After 5 days, 6.25 µM OXA significantly inhibited cell viability, when compared to sham-treated cells; however, 5 mg/mL PRIVIGEN^®^ IgG significantly reverted these toxic effects of oxaliplatin. The green dots indicate cells treated by sham, the red triangles indicate cells treated by oxaliplatin, the black dot indicates cells treated by IgG and the white dots indicate cells treated by oxaliplatin plus IgG. $ indicates *P*-values determined by Student *t* test; * indicates *P*-values determined by Mann–Whitney *U* test. *N* = 9 for (**a**–**h**); *N* = 8 for (**l**); *N* = 7 for (**k**); *N* = 6 for (**i**) and (**j**).

Next, the anti-carcinoma effects of classical drugs to treat colon cancer, i.e., 5-fluorouracil, irinotecan and oxaliplatin, were analysed in the presence and absence of 5 mg/mL PRIVIGEN^®^ IgG. HROC cell lines were sensitive to 5-fluorouracil (data not shown), irinotecan (data not shown) and oxaliplatin when cell viability was evaluated in the absence or in the presence of escalating doses of these drugs for 5 days. Oxaliplatin significantly inhibited the viability of HROC cells in a dose-dependent manner (Fig. 1; 1 µM, 2.5 µM, 6.25 µM and 15.6 µM oxaliplatin). When combining PRIVIGEN^®^ with 5-fluorouracil and irinotecan, no effect of IgG on the anti-carcinoma activity of the cytotoxic drugs was observed (data not shown). The same negative result was obtained after incubation of colon cancer cells with IgG and 6.25 µM oxaliplatin for 1 day (Fig. 2a–d) and 3 days (Fig. 2e–h). However, after a longer incubation time of 5 days, the anti-viability effect of oxaliplatin (Fig. 2i–l, red triangles indicating cells treated by oxaliplatin) was significantly impaired by the addition of 5 mg/mL PRIVIGEN^®^ IgG in all four cell lines (Fig. 2i–l, white dots indicate cells treated by the combinational therapy). Sham-treated HROC277, HROC285, HROC370 and HROC374 cells are shown as a reference (Fig. 2i–l, green dots).

**Fig. 3:**
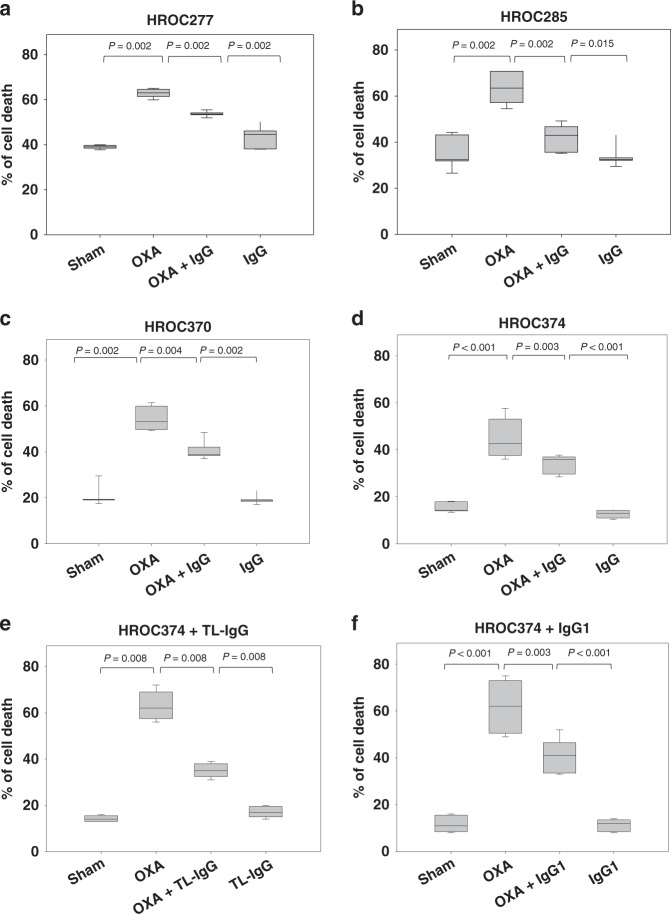
Pharmaceutical IgG decreases oxaliplatin-induced cell death. HROC277 (**a**), HROC285 (**b**), HROC370 (**c**) and HROC374 (**d**) cells were incubated with sham, 6.25 µM oxaliplatin (OXA), 5 mg/mL PRIVIGEN^®^ IgG, 5 mg/mL Tonglu^®^ IgG (**e**), 100 ng/mL IgG1 (**f**) or OXA in combination with IgG for 48 h. In all, 6.25 µM OXA significantly induced cell death, when compared to sham-treated cells. In the combinatorial therapy, a significant decrease in this OXA-triggered cell death was observed. The *P*-values were determined by Mann–Whitney *U* test. *N* = 11 for **d**; *N* = 6 for (**a**), (**b**) and (**c**); *N* = 5 for (**e**) and (**f**).

Subsequently, cell death was evaluated in more detail using the classical Annexin V/PI staining technique. In line with the previous results, 6.25 µM oxaliplatin significantly induced cell death of HROC277, HROC285, HROC370 and HROC374 cells, when compared to sham-treated cells (Fig. 3a–d). Again, the combinational therapy (5 mg/mL IgG plus 6.25 µM oxaliplatin) significantly reduced cell death when compared to oxaliplatin-treated cells (Fig. 3a–d). These data suggest that PRIVIGEN^®^ IgG, the U.S. Food and Drug Administration approved human IgG, at least under experimental conditions, impairs the anti-carcinoma activity of oxaliplatin.

In order to evaluate if other pharmaceutical IgG also inhibited the cell toxicity of oxaliplatin, we incubated HROC374 cells with 6.25 µM oxaliplatin and 5 mg/mL Tonglu^®^ IgG, which was approved by the Chinese National Medical Products Administration. Similar to PRIVIGEN^®^ IgG, Tonglu^®^ IgG did not significantly influence cell death, when compared to sham-treated cells (Fig. 3e). However, it significantly reduced the oxaliplatin-induced cell death, when compared to oxaliplatin-treated cells (Fig. 3e). Due to the fact that PRIVIGEN^®^ IgG mainly contained IgG1 (61%), we thus additionally evaluated if IgG1 contributed to the inhibition of the anticancerous activity of oxaliplatin. Indeed, also IgG1 alone significantly decreased oxaliplatin-induced colon cancer cell death (Fig. 3f).

Overall, these data suggest that the cell toxicity of oxaliplatin was significantly reduced by human normal IgG, including pharmaceutical IgG such as PRIVIGEN^®^ IgG and Tonglu^®^ IgG, and that IgG1 at least contributes to this surprising effect.

### HROC cancer cell lines have rearranged IgG gene loci and produce tumour-derived IgG

Subsequently, we evaluated if cell lines of the low-passaged HROC collection could generate IgG. Indeed, the levels of intrinsic IgG in HROC cells were significantly higher than in T cells used as negative control cells (Supplementary Fig. 3A). However, the IgG amount secreted into the medium by the HROC cells was very low (Supplementary Fig. 3B). This suggests that, while colon cancer cells are capable of generating IgG, they, contrary to the normal physiological behaviour of B cells, retain this IgG intracellularly. In order to support the observation that HROC cells can generate IgG by mechanistical data, we additionally determined the rearrangements of the IgG gene loci in four of the cell lines: HROC370, HROC374, HROC285 and HROC277. We observed monoclonal rearrangement of the heavy chain of IgG (IgH, Supplementary Fig. 3C) and the light chain of IgG (IgK, Supplementary Fig. 3D), which is a prerequisite for IgG production. In addition, we observed that the levels of this tumour cell-derived IgG positively correlate with the IC_50_ of oxaliplatin (Pearson’s correlation coefficient = 0.643, *P* = 0.022, data not shown).

### Pharmaceutical IgG impairs oxaliplatin-activated ERK1/2 signal transduction

**Fig. 4:**
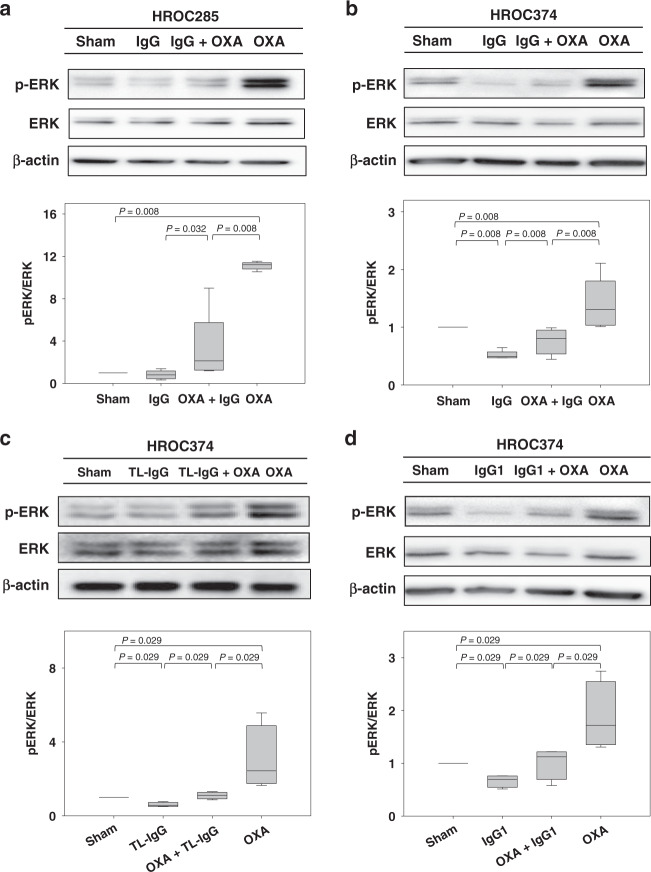
Pharmaceutical IgG impairs oxaliplatin-induced ERK1/2 signal transduction. Colon cancer cells were treated with 6.25 µM oxaliplatin (OXA), 5 mg/mL PRIVIGEN^®^ IgG (**a**, **b**), Tonglu^®^ IgG (TL-IgG, **c**), IgG1 (**d**) or an OXA-containing combination for 36 h. We observed that OXA markedly activated ERK1/2, whereas PRIVIGEN^®^ IgG, TL-IgG and IgG1 impaired ERK1/2 phosphorylation. The *P*-values were determined by Mann–Whitney *U* test. *N* = 5 for (**a**), (**b**) and (**d**); *N* = 4 for (**c**).

In order to shed light on the mechanism(s) of interaction between the pharmaceutical IgG and oxaliplatin, we evaluated the levels of ERCC1 and p-ERK1/2, two proteins that might be involved in the cell toxicity of oxaliplatin.^10–15^ After incubation of colon cancer cells with 5 mg/mL PRIVIGEN^®^, 6.25 µM oxaliplatin or the combinatorial therapy, we observed that both IgG and oxaliplatin had no effect on the accumulation of ERCC1 (Supplementary Fig. 4). Interestingly, oxaliplatin significantly induced a strong phosphorylation of ERK1/2 (*P* = 0.008, Fig. 4a), whereas PRIVIGEN^®^ IgG reduced the phosphorylated ERK1/2 concentration in HROC285 cells (Fig. 4a). A similar strong activation of the ERK1/2-signalling pathway by oxaliplatin and a significant downregulation of this signalling pathway by IgG could be validated in HROC374 cells (Fig. 4b). In addition, after incubating HROC374 cells with Tonglu^®^ IgG or IgG1, we also observed an inhibition of the oxaliplatin-mediated phosphorylation of ERK1/2 (Fig. 4c, d).

**Fig. 5:**
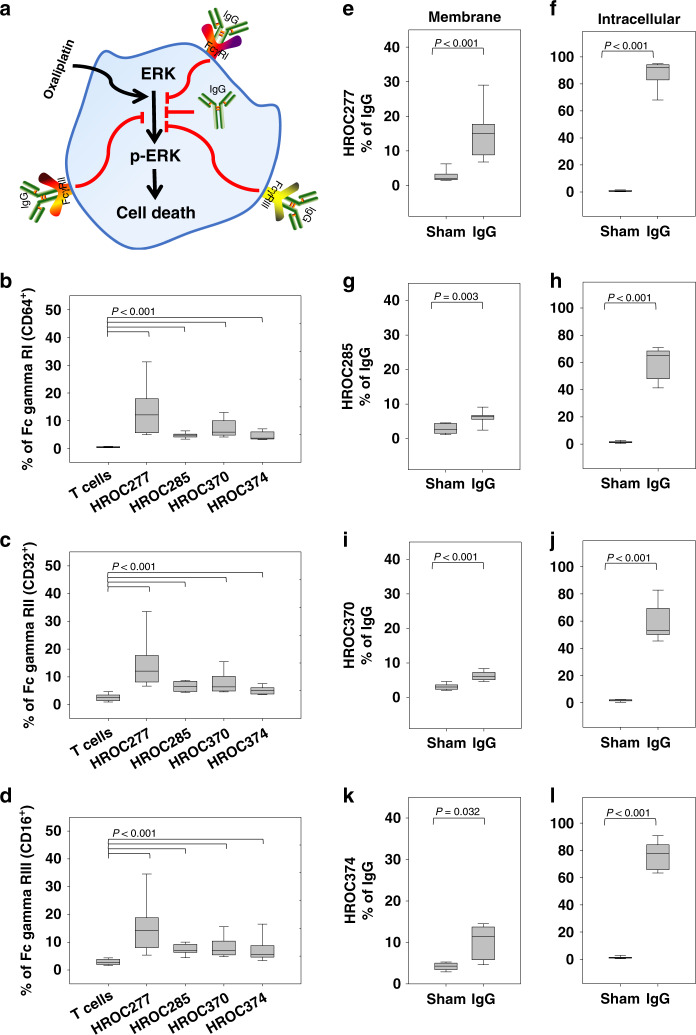
PRIVIGEN^®^ IgG not only binds to the receptors on the surface of colon cancer cells but is also internalised. Three receptors of IgG, FcγRI, FcγRII and FcγRIII (**a**) were analysed in T cells, HROC277, HROC285, HROC370 and HROC374. Compared to T cells, the colon cancer cells expressed a significantly higher level of FcγRI (**b**), FcγRII (**c**) and FcγRIII (**d**). In addition, after incubation with PRIVIGEN^®^ IgG for 24 h, we observed that PRIVIGEN^®^ IgG not only binds to the receptors (**e**, **g**, **i** and **k**), it is also internalised by cancer cells (**f**, **h**, **j** and **l**). Of note, cells stained without PRIVIGEN incubation and thus displaying the intrinsic and cancer cell-derived IgG of the respective HROC cells (individual levels can be depicted from Supplementary Fig. 3) were set as the blank reference. The *P*-values were determined by Mann–Whitney *U* test. *N* = 9 for (**e**), (**f**), (**h**), (**j**), (**l**); *N* = 8 for (**b**–**d** and **g**); *N* = 7 (**i**); *N* = 5 for (**k**).

In order to investigate if these IgG effects are mediated via binding to receptors on the surface of colon cancer cells or if the IgG is internalised by the colon cancer cells (Fig. 5a), we determined first the expression levels of FcγRI, FcγRII and FcγRIII, the three major receptors of IgG.^28^ In comparison to the negative control, human peripheral T cells, the levels of FcγRI (Fig. 5b), FcγRII (Fig. 5c) and FcγRIII (Fig. 5d) were significantly higher in all four colon cancer cell lines analysed. Secondly, the colon cancer cells were incubated with PRIVIGEN^®^ IgG before the levels of membrane-bound and intracellular IgG were evaluated, respectively. Compared to sham-treated cells, the PRIVIGEN^®^ pre-incubation significantly increased the levels of both membrane-bound and intracellular IgG in HROC277 (Fig. 5e, f), HROC285 (Fig. 5g, h), HROC370 (Fig. 5i, j) and HROC374 cells (Fig. 5k, l). This suggests that the IgG-mediated inhibition of ERK1/2 activity might depend on receptor binding, but a contribution of internalised IgG is also possible.

### The anti-carcinoma activity of oxaliplatin is dependent on ERK1/2 activity

**Fig. 6:**
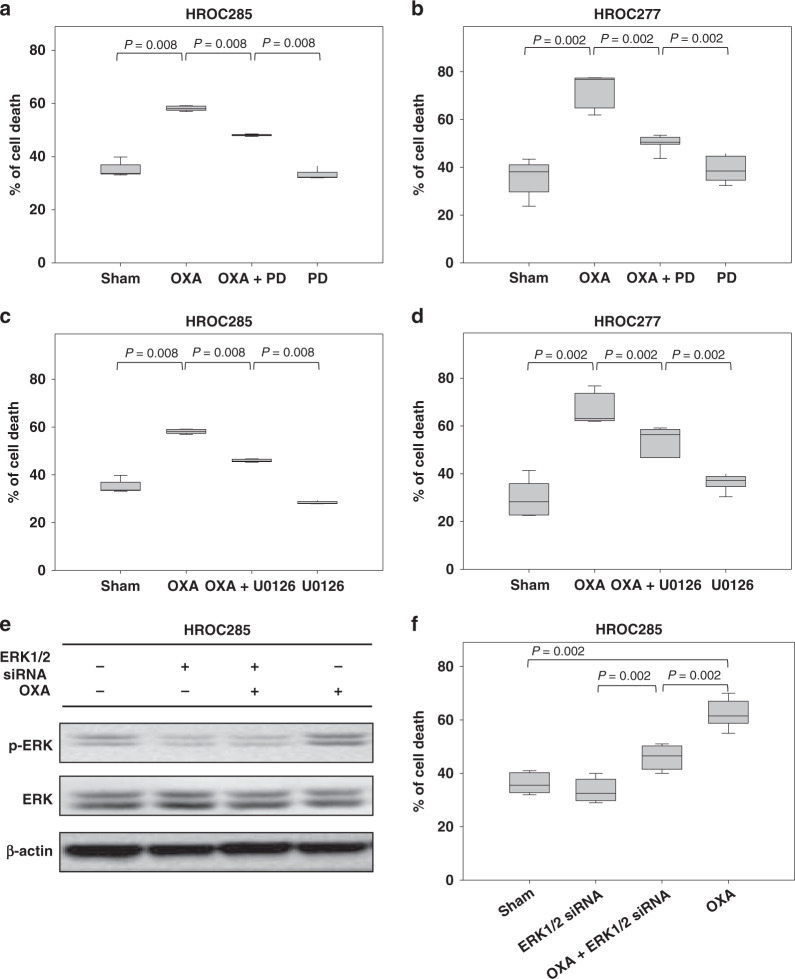
The anti-carcinoma activity of oxaliplatin is dependent on the ERK1/2 activity. Colon cancer cells (HROC285, HROC277) were pretreated with 50 µM PD98059 (**a**, **b**) or 10 µM U0126 (**c**, **d**) for 1 h. Subsequently, these cells were treated by oxaliplatin (OXA) for 48 h. OXA significantly increased the percentage of dead cells, whereas the ERK1/2 inhibitors PD98059 (PD) or U0126 significantly decreased OXA-induced cell death. Moreover, inhibition of the ERK1/2 activity by ERK1/2 siRNA (**e**) could significantly impair the oxaliplatin-induced cell death (**f**) too. The *P-*value was determined by the Mann–Whitney *U* test. *N* = 5 for (**a**), (**c**) and (**d**); *N* = 7 for (**b**); N = 3 for (**e**); *N* = 6 for (**f**).

In order to clarify if and how ERK1/2 was involved in the oxaliplatin-mediated inhibition of cell viability, HROC285 cells were treated with 50 µM PD98059, a traditional inhibitor of ERK1/2. Only a minor influence on control cells (Fig. 6a) was observed, but a significant inhibition of oxaliplatin-induced cell death (*P* = 0.008, Fig. 6a) occurred in PD98059-treated cells. Similar results were obtained when treating HROC277 cells with sham, 6.25 µM oxaliplatin, 50 µM PD98059 or the combination therapy (*P* = 0.002, Fig. 6b). These findings could be reproduced using U0126, another inhibitor of ERK1/2 to treat HROC285 (*P* = 0.008, Fig. 6c) and HROC277 cells (*P* = 0.002, Fig. 6d). Inhibiting ERK1/2 activity by p44/42 (ERK1/2) MAP kinase siRNA (Fig. 6e) did not significantly decrease cell death. But again, this siRNA significantly decreased the level of oxaliplatin-induced cell death in the combinatorial therapy, when compared to the oxaliplatin-treated cells (*P* = 0.002, Fig. 6f). These data suggest that the anti-carcinoma activity of oxaliplatin is at least partially dependent on ERK1/2 activity.

## Discussion

The present study, for the first time, demonstrates that human normal IgG can inhibit the anti-carcinoma activity of the classical chemotherapeutic oxaliplatin. Few studies demonstrated that intravenous IgG can inhibit tumour growth,^19–22,30^ and it had been suggested that this effect might depend on the immunological activity of IgG. For example, Carmi et al. reported that IgG could recognise surface antigens on tumour cells and bind to the Fcγ receptor on dendritic cells. When the dendritic cells are activated, they can present tumour antigens to T cells. Subsequently, these tumour-antigen-specific T cells can eliminate tumour cells.^31^ However, these studies did not evaluate if IgG interferes with the cytotoxic activity of chemical drugs.

Intrinsic as well as induced chemoresistance is a major challenge for clinicians when treating (colon) cancer patients.^32^ Many mechanisms explaining how drug resistance emerges have already been elucidated.^33^ The data presented in this paper add some additional possibilities to explain tumour cells’ evasion potential by a class of molecules previously considered as neutral, in rare cases negative for tumour cells: human immunoglobulins.

Several studies observed that cancer cells from different entities are capable of producing immunoglobulins, especially IgG,^34–36^ and it has even been speculated that this tumour-derived IgG contributes to the development and growth of colon cancer.^37^

The present study not only delivers data to support the previous observation that colon cancer cells possibly regularly but at least frequently are capable of expressing IgG protein(s). Noteworthy, this is the first study to find the gene loci of IgG rearranged, which is even according to textbook knowledge a necessity for IgG production. Potentially of higher importance is the finding that pharmaceutical-grade IgG can inhibit the anticancerous activity of oxaliplatin in several colon cancer cell lines. These data imply that normal IgG may be involved in oxaliplatin resistance. However, this hypothesis clearly needs to be further investigated. In vivo studies, either in well-selected animal models or analyses from fresh tumour explants from colon cancer patients, are here clearly indicated.

One might also speculate that tumour cell-derived IgG has a similar effect on oxaliplatin. The different molecular subtypes of colon cancer have been associated with different survival outcomes and chemotherapy responses.^38,39^ Mesenchymal colon cancer is amongst the subtypes associated with the worst survival outcome.^38,40^ We thus also tried to correlate the level of tumour cell-derived IgG in the HROC cell lines analysed with the molecular features of microsatellite and chromosomal instability as well as the level of CpG island methylation, the three major molecular characteristics of colon cancer cells [data not shown]. To some degree unexpected, no significant correlation was observed, which might, of course, simply be attributable to the still low amount of cases included in this analysis.

A large body of preclinical evidence emphasised that ERCC1, which can inhibit the damage of cellular DNA, might be the most important regulator of resistance to platinum derivatives.^10,41^ In addition, low expression of ERCC1 in clinical samples correlates significantly with longer survival.^42^ However, in the present study, the observation that IgG impaired the cell toxicity of oxaliplatin was not dependent on ERCC1 accumulation.

Instead, the anticancerous activity of oxaliplatin was found to be dependent on the ERK1/2-signalling pathway. Our data are, therefore, apparently contradicting several publications, which demonstrated that ERK1/2 activation can promote tumour growth and inhibit cancer cell apoptosis.^43,44^ However, others demonstrated that the ERK1/2-signalling pathway might also be positively involved in cell apoptosis.^12–15^ In support of the latter findings, inhibition of ERK1/2 activity by pharmaceutical IgG, ERK1/2 siRNA, PD98059 or U0126 impaired oxaliplatin-induced cell death. In order to explain these divergent findings, a context-dependent function of ERK1/2 delivers a likely and logical explanation. Thus, even though a number of preclinical studies suggested inhibition of ERK1/2 activity as a promising treatment option in several cancers^43,45^ with even a clinical trial ongoing (ClinicalTrials.gov Identifier: NCT02420795), the data of the present study advice caution for such strategies. Mode of action and potential benefits of ERK1/2 inhibitors should be fully investigated preclinically before clinical application in cancer patients’ treatment.

In conclusion, this study demonstrates that clinically applied IgG therapeutics consisting of pooled normal human IgG, could directly impair the anti-carcinoma activity of oxaliplatin in colon cancer cells. Mechanistically, this effect of IgG might be due to impairment of the ERK1/2 signal transduction pathway. Hence, these data urge caution for clinical application of both pharmaceutical IgG as well as ERK1/2 inhibitors in the context of oxaliplatin-containing anticancer therapies.

## Supplementary information

Supplementary Figures

## Data Availability

The data and materials are available from the corresponding author on reasonable request.
