# Supplementary material for: Pharmaceutical immunoglobulin G impairs anti-carcinoma activity of oxaliplatin in colon cancer cells
Source: Br J Cancer. 2021 Feb 9;124(8):1411–20. doi: 10.1038/s41416-021-01272-6 (PMC8039037; doi:10.1038/s41416-021-01272-6)
Supplement: Supplementary file 1 — Supplementary Figures [file 41416_2021_1272_MOESM1_ESM.pptx]

## Slide 1
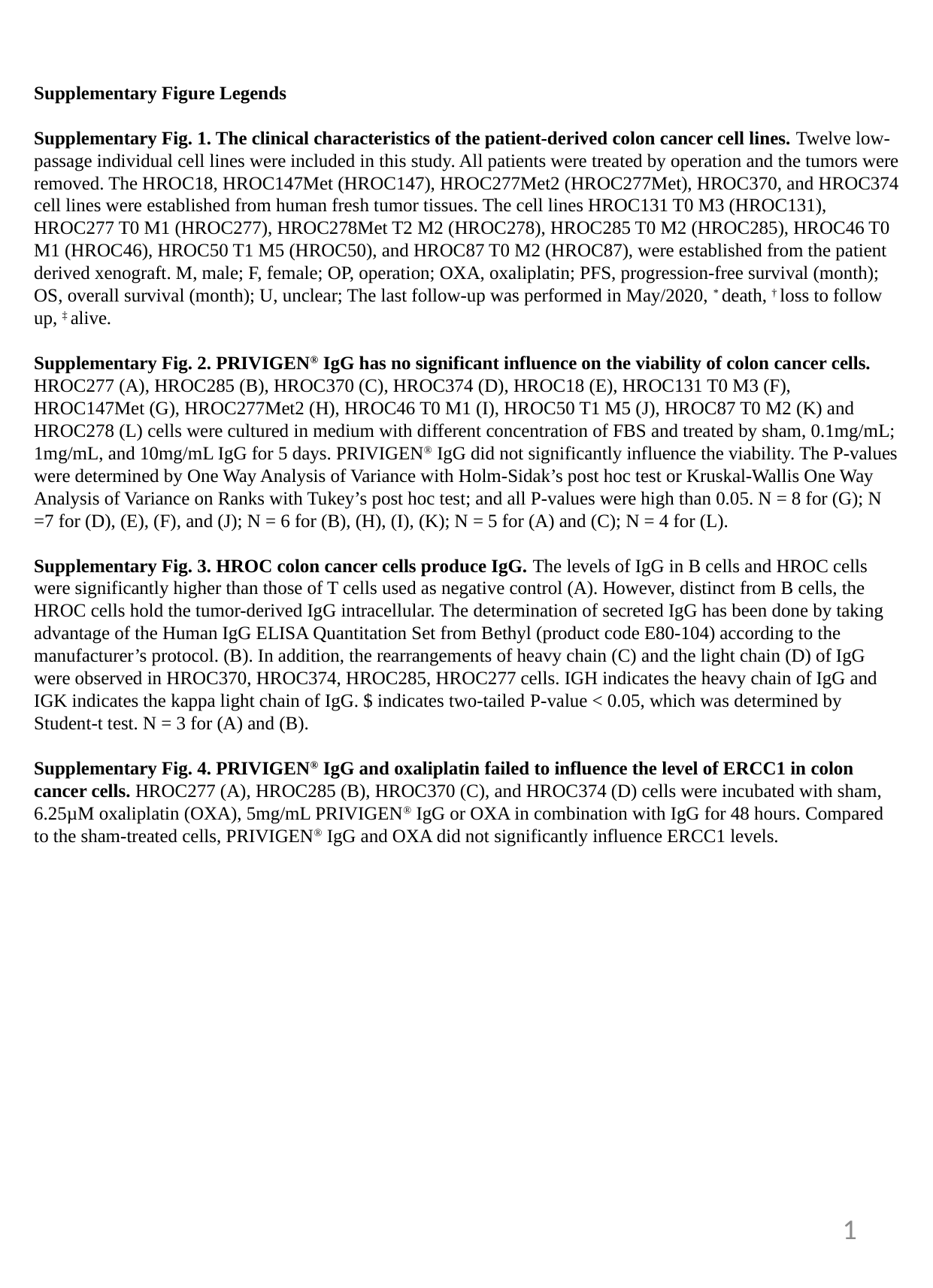

Supplementary Figure Legends
Supplementary Fig. 1. The clinical characteristics of the patient-derived colon cancer cell lines. Twelve low-passage individual cell lines were included in this study. All patients were treated by operation and the tumors were removed. The HROC18, HROC147Met (HROC147), HROC277Met2 (HROC277Met), HROC370, and HROC374 cell lines were established from human fresh tumor tissues. The cell lines HROC131 T0 M3 (HROC131), HROC277 T0 M1 (HROC277), HROC278Met T2 M2 (HROC278), HROC285 T0 M2 (HROC285), HROC46 T0 M1 (HROC46), HROC50 T1 M5 (HROC50), and HROC87 T0 M2 (HROC87), were established from the patient derived xenograft. M, male; F, female; OP, operation; OXA, oxaliplatin; PFS, progression-free survival (month); OS, overall survival (month); U, unclear; The last follow-up was performed in May/2020, * death, † loss to follow up, ‡ alive.
Supplementary Fig. 2. PRIVIGEN® IgG has no significant influence on the viability of colon cancer cells. HROC277 (A), HROC285 (B), HROC370 (C), HROC374 (D), HROC18 (E), HROC131 T0 M3 (F), HROC147Met (G), HROC277Met2 (H), HROC46 T0 M1 (I), HROC50 T1 M5 (J), HROC87 T0 M2 (K) and HROC278 (L) cells were cultured in medium with different concentration of FBS and treated by sham, 0.1mg/mL; 1mg/mL, and 10mg/mL IgG for 5 days. PRIVIGEN® IgG did not significantly influence the viability. The P-values were determined by One Way Analysis of Variance with Holm-Sidak’s post hoc test or Kruskal-Wallis One Way Analysis of Variance on Ranks with Tukey’s post hoc test; and all P-values were high than 0.05. N = 8 for (G); N =7 for (D), (E), (F), and (J); N = 6 for (B), (H), (I), (K); N = 5 for (A) and (C); N = 4 for (L).
Supplementary Fig. 3. HROC colon cancer cells produce IgG. The levels of IgG in B cells and HROC cells were significantly higher than those of T cells used as negative control (A). However, distinct from B cells, the HROC cells hold the tumor-derived IgG intracellular. The determination of secreted IgG has been done by taking advantage of the Human IgG ELISA Quantitation Set from Bethyl (product code E80-104) according to the manufacturer’s protocol. (B). In addition, the rearrangements of heavy chain (C) and the light chain (D) of IgG were observed in HROC370, HROC374, HROC285, HROC277 cells. IGH indicates the heavy chain of IgG and IGK indicates the kappa light chain of IgG. $ indicates two-tailed P-value < 0.05, which was determined by Student-t test. N = 3 for (A) and (B).
Supplementary Fig. 4. PRIVIGEN® IgG and oxaliplatin failed to influence the level of ERCC1 in colon cancer cells. HROC277 (A), HROC285 (B), HROC370 (C), and HROC374 (D) cells were incubated with sham, 6.25µM oxaliplatin (OXA), 5mg/mL PRIVIGEN® IgG or OXA in combination with IgG for 48 hours. Compared to the sham-treated cells, PRIVIGEN® IgG and OXA did not significantly influence ERCC1 levels.
1

## Slide 2
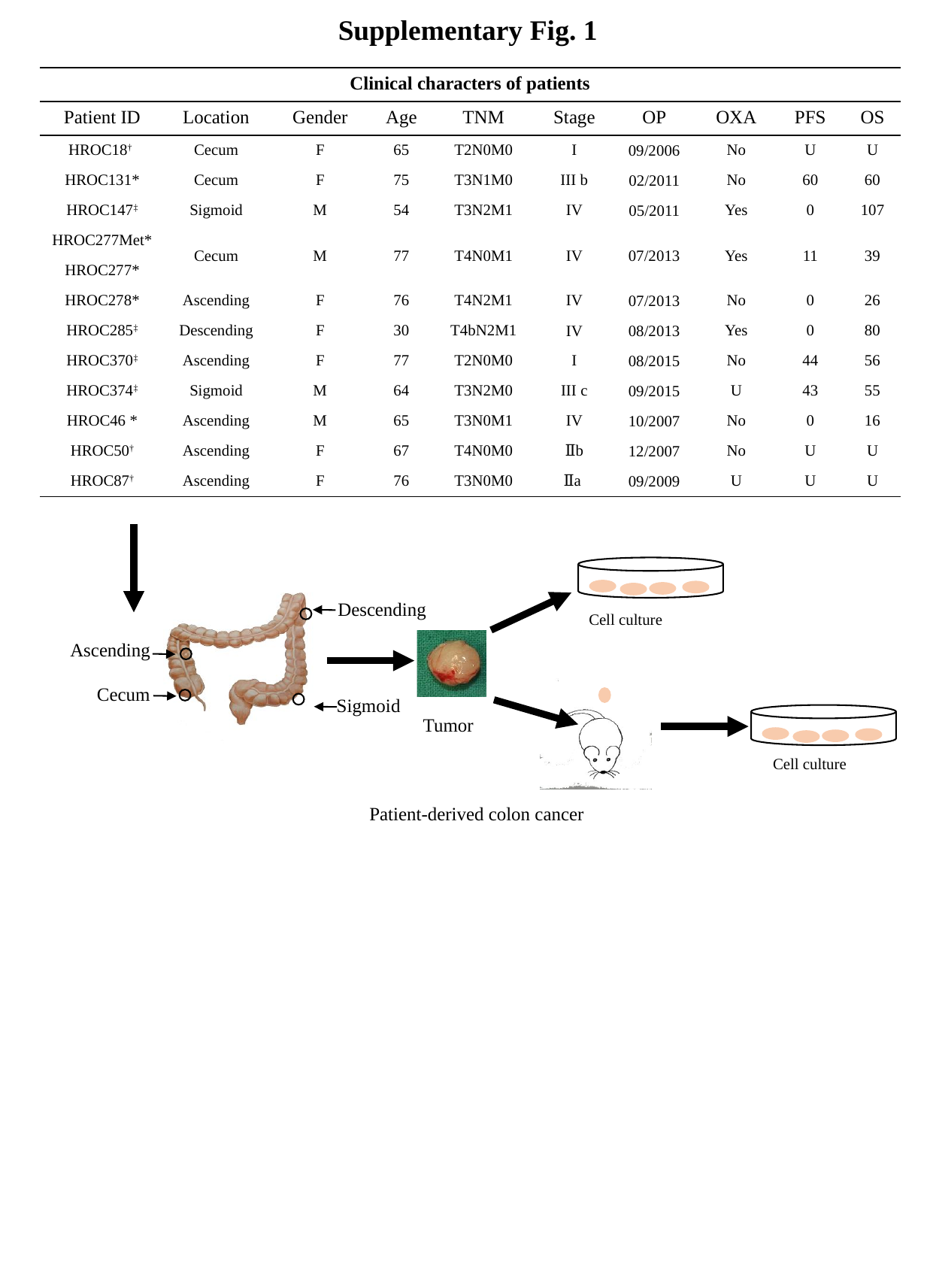

Supplementary Fig. 1
| Clinical characters of patients | | | | | | | | | |
| --- | --- | --- | --- | --- | --- | --- | --- | --- | --- |
| Patient ID | Location | Gender | Age | TNM | Stage | OP | OXA | PFS | OS |
| HROC18† | Cecum | F | 65 | T2N0M0 | I | 09/2006 | No | U | U |
| HROC131\* | Cecum | F | 75 | T3N1M0 | III b | 02/2011 | No | 60 | 60 |
| HROC147‡ | Sigmoid | M | 54 | T3N2M1 | IV | 05/2011 | Yes | 0 | 107 |
| HROC277Met\* | Cecum | M | 77 | T4N0M1 | IV | 07/2013 | Yes | 11 | 39 |
| HROC277\* | | | | | | | | | |
| HROC278\* | Ascending | F | 76 | T4N2M1 | IV | 07/2013 | No | 0 | 26 |
| HROC285‡ | Descending | F | 30 | T4bN2M1 | IV | 08/2013 | Yes | 0 | 80 |
| HROC370‡ | Ascending | F | 77 | T2N0M0 | I | 08/2015 | No | 44 | 56 |
| HROC374‡ | Sigmoid | M | 64 | T3N2M0 | III c | 09/2015 | U | 43 | 55 |
| HROC46 \* | Ascending | M | 65 | T3N0M1 | IV | 10/2007 | No | 0 | 16 |
| HROC50† | Ascending | F | 67 | T4N0M0 | Ⅱb | 12/2007 | No | U | U |
| HROC87† | Ascending | F | 76 | T3N0M0 | Ⅱa | 09/2009 | U | U | U |
Descending
Cell culture
Ascending
Cecum
Sigmoid
Tumor
Cell culture
Patient-derived colon cancer

## Slide 3
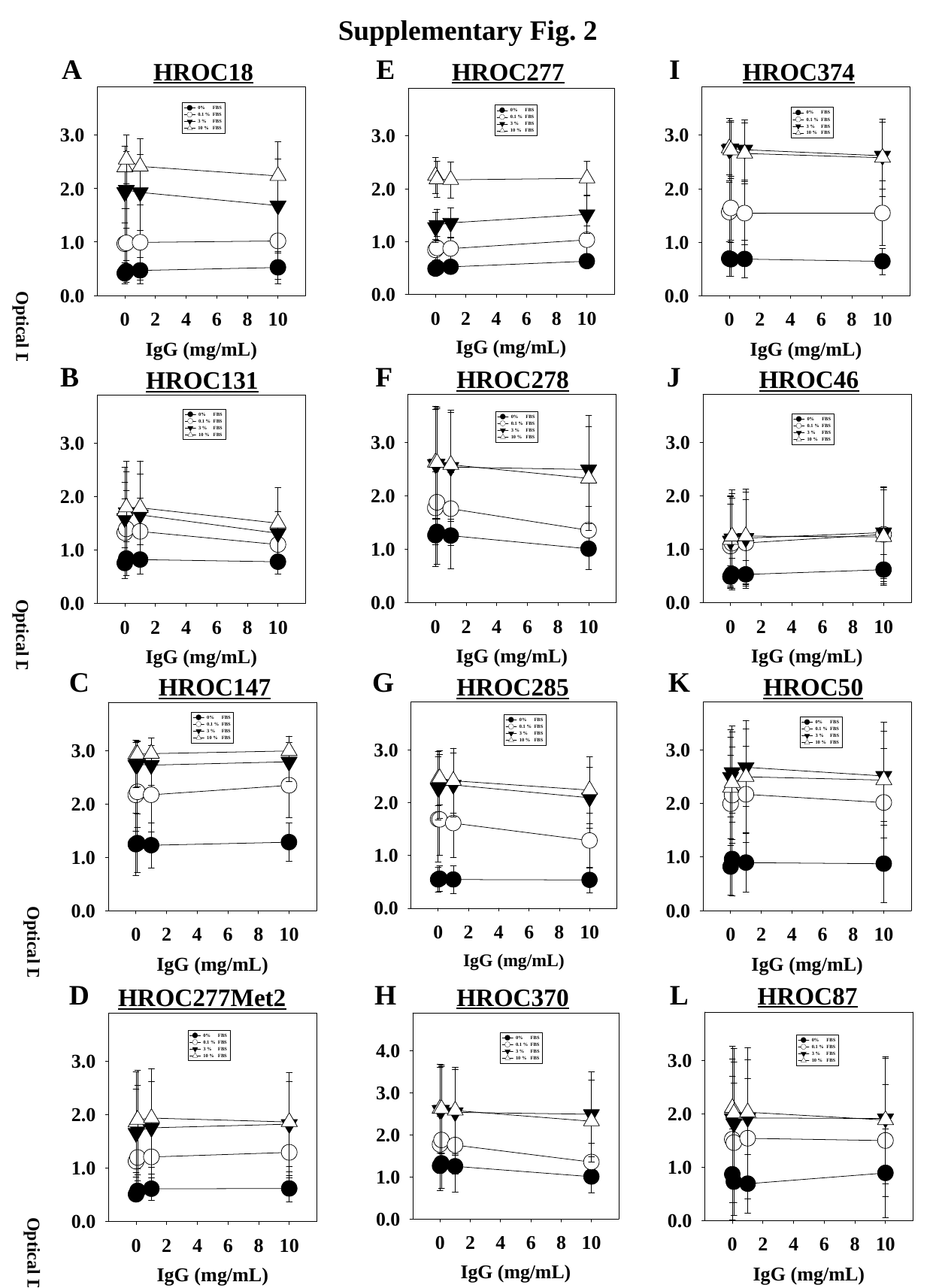

Supplementary Fig. 2
A
E
I
HROC18
HROC277
HROC374
B
F
J
HROC46
HROC278
HROC131
C
G
K
HROC147
HROC285
HROC50
D
H
L
HROC87
HROC370
HROC277Met2

## Slide 4
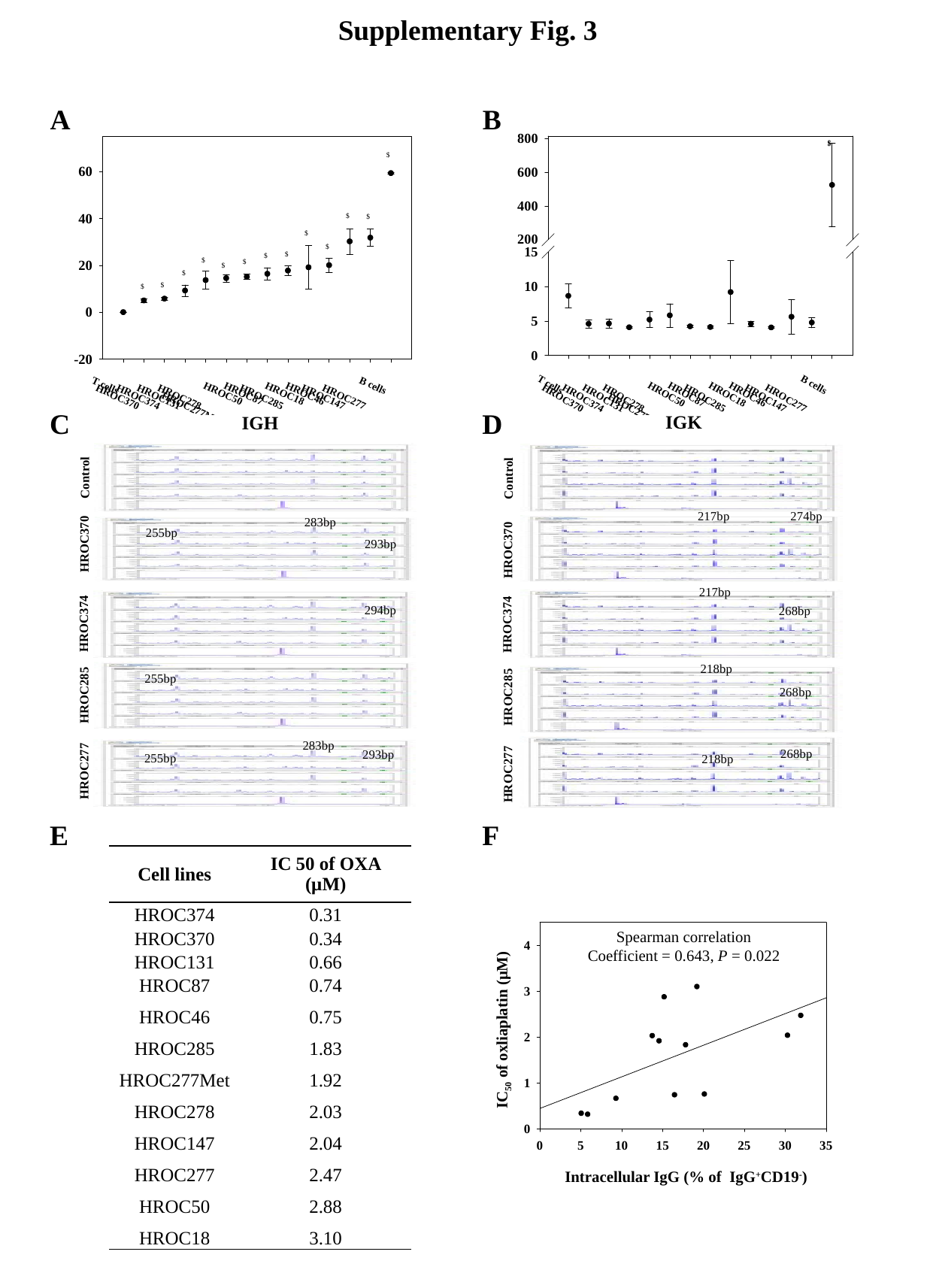

Supplementary Fig. 3
A
B
$
$
$
$
$
$
$
$
$
$
$
$
$
$
$
C
D
IGK
IGH
Control
HROC370
HROC374
HROC285
HROC277
Control
HROC370
HROC374
HROC285
HROC277
274bp
217bp
283bp
255bp
293bp
217bp
294bp
268bp
218bp
255bp
268bp
283bp
268bp
293bp
255bp
218bp
E
F
| Cell lines | IC 50 of OXA (μM) |
| --- | --- |
| HROC374 | 0.31 |
| HROC370 | 0.34 |
| HROC131 | 0.66 |
| HROC87 | 0.74 |
| HROC46 | 0.75 |
| HROC285 | 1.83 |
| HROC277Met | 1.92 |
| HROC278 | 2.03 |
| HROC147 | 2.04 |
| HROC277 | 2.47 |
| HROC50 | 2.88 |
| HROC18 | 3.10 |
Spearman correlation
Coefficient = 0.643, P = 0.022
IC50 of oxliaplatin (μM)
Intracellular IgG (% of IgG+CD19-)

## Slide 5
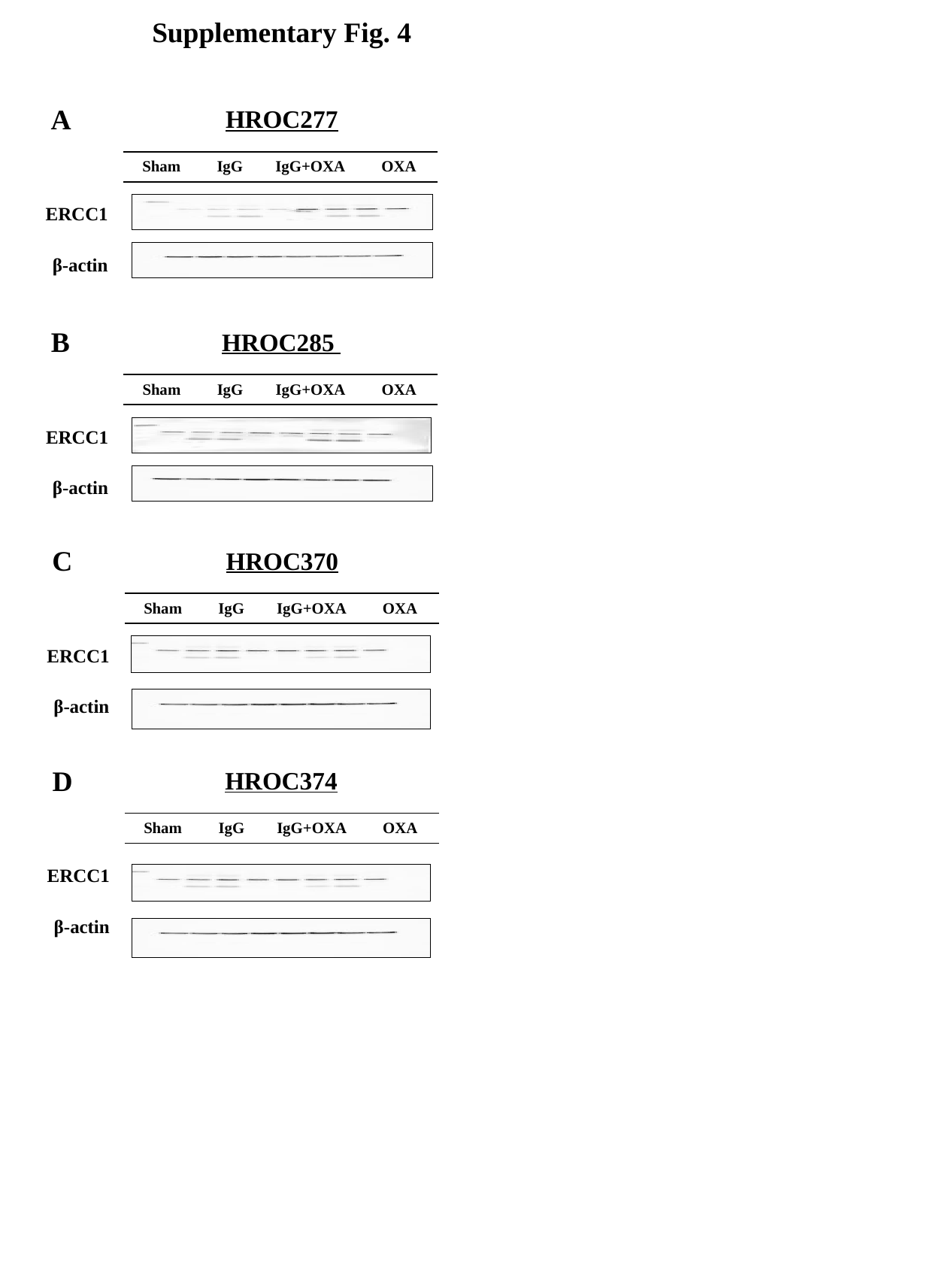

Supplementary Fig. 4
A
HROC277
| Sham | IgG | IgG+OXA | OXA |
| --- | --- | --- | --- |
ERCC1
β-actin
B
HROC285
| Sham | IgG | IgG+OXA | OXA |
| --- | --- | --- | --- |
ERCC1
β-actin
C
HROC370
| Sham | IgG | IgG+OXA | OXA |
| --- | --- | --- | --- |
ERCC1
β-actin
D
HROC374
| Sham | IgG | IgG+OXA | OXA |
| --- | --- | --- | --- |
ERCC1
β-actin
5
